# Protein Supplementation Throughout 10 Weeks of Progressive Run Training Is Not Beneficial for Time Trial Improvement

**DOI:** 10.3389/fnut.2018.00097

**Published:** 2018-11-01

**Authors:** Paul A. Roberson, Matthew A. Romero, Petey W. Mumford, Shelby C. Osburn, Cody T. Haun, Christopher G. Vann, Heidi A. Kluess, Michael D. Roberts

**Affiliations:** ^1^School of Kinesiology, Auburn University, Auburn, AL, United States; ^2^Department of Cell Biology and Physiology, Edward Via College of Osteopathic Medicine, Auburn, AL, United States

**Keywords:** endurance, running, whey protein, NIRS, 5 km time trial, mitochondrial capacity, lean body mass, run training

## Abstract

**Introduction:** Protein supplementation is proposed to promote recovery and adaptation following endurance exercise. While prior literature demonstrates improved performance when supplementing protein during or following endurance exercise, chronic supplementation research is limited.

**Methods:** Runners (VO_2_peak = 53.6 ± 8.9 ml/kg/min) were counter-balanced into a placebo group (PLA; *n* = 8) or protein group (PRO; *n* = 9) based on sex and VO_2_peak, and underwent 10 weeks of progressive endurance training. Prior to training, body composition, blood cell differentials, non-invasive mitochondrial capacity using near-infrared spectroscopy, and a 5 km treadmill time trial (TT) were evaluated. Progressive training then commenced (5–10% increase in weekly volume with a recovery week following 3 weeks of training) whereby PRO supplemented with 25 g of whey protein following workouts and prior to sleep (additional 50 g daily). PLA supplemented similarly with a < 1 g sugar pill per day. Following training, participants were reanalyzed for the aforementioned tests.

**Results:** VO_2_peak and initial 5 km TT were not significantly different between groups. PRO consumed significantly more dietary protein throughout the training period (PRO = 132 g/d or 2.1 g/kg/day; PLA = 84 g/d or 1.2 g/kg/day). Running volume increased significantly over time, but was not significantly different between groups throughout training. Blood measures were unaltered with training or supplementation. Mitochondrial capacity trended toward improving over time (time *p* = 0.063) with no difference between groups. PLA increased lean mass 0.7 kg (*p* < 0.05) while PRO experienced infinitesimal change (−0.1 kg, interaction *p* = 0.049). PLA improved 5 km TT performance 6.4% (1 min 31 s), while PRO improved only 2.7% (40 s) (interaction *p* = 0.080).

**Conclusion:** This is the first evidence to suggest long-term protein supplementation during progressive run training is not beneficial for runners.

## Introduction

Protein supplementation has been used to assist in recovery from exercise as well as increase caloric and protein intake. Current dietary protein recommendations for endurance athletes range from 1.2 to 2.0 g/kg/day (1, 2). Specifically, Kato et al. (3) has suggested intakes of 1.5–1.8 g/kg/day are optimal for endurance athletes, and further demonstrated the primary limiting amino acids are the branched chain amino acids (BCAAs) (4). There is a general consensus that dietary protein is important for refueling, skeletal muscle recovery, and adaptation following endurance exercise (5), especially given amino acids can contribute as high as 20% of total energy yield during exhaustive exercise (6, 7). Additionally, leucine oxidation is increased with endurance exercise (8–10) limiting its contribution to normal anabolic and recovery processes in skeletal muscle (11). Thus, post-exercise protein supplementation seemingly provides an opportunity to mitigate muscle damage and enhance recovery in endurance athletes.

Protein ingestion during endurance exercise has been shown to improve performance (12–14). Following endurance exercise, protein ingestion has also been shown to improve muscle glycogen re-synthesis (15, 16) and successive performance (17, 18). Although acute protein ingestion has shown positive benefits, data regarding long term protein supplementation over weeks to months in endurance athletes is lacking. One of the longest studies to date examining protein supplementation in endurance athletes utilized an intensified cycling training period consisting of a 10-day increased volume mesocycle that was 220% above normal training (19). Interestingly, 30 km time trial (TT) performance was unaffected by protein supplementation compared to carbohydrate supplementation, however muscle quality measured by peak torque and fiber cross sectional area were improved with supplementation. While not a direct supplementation comparison, Kephart et al. (20) demonstrated that 10 weeks of cycle training with 12 g/d of BCAA supplementation improved relative mean power 4% and 4 km TT performance 11%.

Given the improvements from protein supplementation on muscle quality measures, the eccentric contractions and weight-bearing nature of running compared to cycling could better unveil the ergogenic potential of protein supplementation. In this regard, Luden et al. (21) utilized NCAA Division I cross-country runners and supplemented them with a carbohydrate-protein-antioxidant beverage during 6 days of training leading into competition. While plasma creatine kinase and muscle soreness were significantly improved following supplementation, 5 and 8 km (female and male, respectively) TT performance were unaffected by supplementation. Hansen et al. (22) conducted a similar experiment in elite runners participating in a strenuous 1-week training camp, and similar to Luden et al. (21), plasma creatine kinase was significantly lower as the week progressed when supplementing with protein than with carbohydrate. However, in this study, 4 km TT performance improved in the carbohydrate-protein group while performance in the carbohydrate group worsened.

The discrepant findings on protein supplementation in endurance athletes in tandem with the lack of long-term training studies led us to examine if protein supplementation could affect physiological and performance variables in runners during 10 weeks of progressive run training. We hypothesized protein supplementation would improve body composition and 5 km TT performance compared to a non-isocaloric placebo based on the anabolic and recovery properties associated with protein supplementation and/or the addition of energy (kcal).

## Materials and methods

### Participants

This study was approved by the Auburn University Institutional Review Board and was in compliance with the Helsinki Declaration (IRB protocol: #17-231 MR 1706). Participants read and signed an informed consent form prior engaging in the study. Inclusion criteria were as follows: (a) participants could be males or females between the ages of 18–45 years old, (b) participants had to partake in at least 32 km (~20 mi) per week of run training for at least one month prior to the study, (c) participants had to be healthy and free of any known disease determined by medical history questionnaire, and (d) participants had to abstain from supplemental protein or amino acids for 3 months prior to participating. A physical activity questionnaire and medical history form were filled out prior to participation to establish physical activity requirements were met and to identify potential risk factors that could be aggravated by training.

### Experimental design

Participants completed an initial session consisting of a VO_2_peak test and 5 km time trial (TT) familiarization. Participants were then counter-balanced based on sex and VO_2_peak into a placebo group (PLA) or protein group (PRO). Pretesting was performed 48 h later and consisted of: (a) dual-energy x-ray absorptiometry (DEXA), (b) venipuncture from an antecubital vein for blood cell measurements, (c) mitochondrial capacity measurement using near-infrared spectroscopy (NIRS), and (d) 5 km treadmill TT performance. At the conclusion of pretesting, participants were given canisters of protein powder or canisters of placebo sugar pills (described under “Supplementation and Food Logs”). Notably, this study was single-blinded and participants were informed that either supplement may be more beneficial based on the sparse chronic protein supplementation literature in endurance athletes.

### VO_2_peak testing

VO_2_peak testing was performed to determine maximal aerobic capacity. Participants reported to all testing sessions not having consumed alcohol in 24 h, chewed/smoked tobacco in 24 h, consumed caffeine in 8 h, consumed calories in 4 h, or engaged in strenuous exercise for 36 h. Participants were then fitted with a Polar heart rate monitor (Lake Success, NY, USA) and mounted a Woodway Treadmill (Waukesha, WI, USA). Following mouthpiece and headgear fitting, expired gases were continuously analyzed using a ParvoMedics TrueMax 2400 metabolic measurement system (Sandy, UT, USA). Participants walked at 1.34 m/s (3.0 mi/h) for 3 min and then lightly jogged at 2.46 m/s (5.5 mi/h) for 2 min for a warm-up. Participants' speed was then increased to a pace which he/she deemed suitable for a 30-min run, and grade was increased to 1% to start the test. Grade was increased 1% every 1 min until volitional fatigue. Heart rate (HR), rating of perceived exertion (RPE), and respiratory exchange ratio (RER) were recorded every minute until cessation of the test. A test was accepted if heart rate was within 5% of age-predicted max HR, RPE ≥ 17, and RER ≥ 1.05. Average age for females and males was 30.9 ± 10.3 and 31.0 ± 7.2 years old, respectively. Average VO_2_peak for females and males was 47.0 ± 4.2 and 58.1 ± 8.5, respectively. Based on average age and values put forth by the American College of Sports Medicine (23), both the females and males ranked above the 90th and 95th percentiles, respectively. Participants' VO_2_peak value was used to counter-balance supplementation groups as group designation was determined prior to pre 5 km performance and potential trainability to the running program based on the VO_2_peak value (i.e., those with a lower VO_2_peak value would respond to training greater than those with higher a VO_2_peak value).

### Familiarization

Following VO_2_peak testing, participants were familiarized for the 5 km TT. Participants mounted the treadmill and were instructed to run 5 km (3.11 mi) as fast as possible. Participants were blinded to time and speed in order to avoid motivation from these variables and practiced starting the treadmill under these conditions. Once comfortable, grade was placed at 1%, and the TT began once the participant increased the treadmill speed. Upon starting, participants raised the speed until a self-selected pace was achieved which could then be manipulated throughout the TT based on desire to run faster or slower. Participants were monitored throughout the TT, but no verbal encouragement was given from the research staff. Once participants reached 5 km, time to completion was recorded, and participants walked for 5 min at a self-selected pace to cool-down.

### Testing procedures

Forty eight to seventy two hours following VO_2_peak testing and familiarization, testing began with assessment of hydration status measured through urine testing by an ATAGO 2392 handheld refractometer (Bellvue, WA, USA). Participants were considered hydrated if urine specific gravity level was < 1.020 g/ml which all participants met. Following hydration testing, participants underwent a dual-energy X-ray absorptiometry (DEXA) scan on a GE Corporation Lunar Prodigy (Fairfield, Connecticut, USA) to determine body composition. Reliability for this test has been previously reported by our laboratory (intraclass correlation coefficient = 0.999) (20). Thereafter, participants laid supine and rested for 10 min prior to an antecubital venous blood draw. Blood was drawn into a 4 ml EDTA tube (BD Vacutainer, Franklin Lakes, NJ, USA), transported to the CLIA certified Auburn University Medical Clinic following attainment, and complete blood count (CBC) panels were analyzed using Beckman Coulter DxH 600 Hematology analyzer (Beckman Coulter, Fullerton, CA, USA). During the 10 min resting period participants were set-up for non-invasive mitochondrial capacity assessment using near-infrared spectroscopy (NIRS). Briefly, participants were fitted for the NIRS optode (Artinis Medical System, Oxymon MKIII, Elst, The Netherlands) on the medial gastrocnemius around the largest circumference of the calf via self-adhering straps. The gastrocnemius was utilized given the stress and demand running specifically places on the gastrocnemius. Electrical stimulation pads were placed proximally and distally to the NIRS device to stimulate contraction of the gastrocnemius (Grass Medical Instruments, Quincy, MA, USA), and stimulation intensity was increased until contraction was visually seen (participants were not uncomfortable or in pain during stimulation). A medium-sized blood pressure cuff connected to a rapid cuff inflator (Hokanson Inc., Bellevue, WA, USA) was then placed just distally to the patella and inflated to 240 mmHg throughout the protocol to occlude blood flow. Participants underwent a series of blood pressure cuff oscillations and electrical stimulations to determine mitochondrial capacity. These data were analyzed in MATLAB (The MathWorks Inc., Natick, MA, USA) and a Portamon Analysis System (US Patent #9,706,959). Time constants were generated using slopes from the arterial blood occlusions to create a curve from the averaged oxygenated and deoxygenated blood signal, and data were reported as a rate constant (min^−1^). Detailed set-up, mitochondrial capacity assessment using NIRS procedures, analysis, and reliability of the device have been detailed elsewhere (24, 25). At the conclusion of the mitochondrial capacity assessment, participants completed a warm-up and 5 km TT performance identical to the familiarization session which has been demonstrated to be a reliable measure (26), especially following a familiarization (27). Time was recorded to the nearest second. Of note, female participants were not controlled for menstrual cycle. While female steroid hormones can affect metabolism (28), their impact on performance metrics is mixed (29). Following 10 weeks of progressive run training with supplementation, participants returned and were retested for the aforementioned measures. Participants were blinded to all pretesting data until all testing sessions were complete.

### Training

Once pretesting concluded participants began run training for 10 weeks. All participants started the same Sunday, and a training week was from Sunday to Saturday. Participants were instructed to meet a recommended weekly volume (distance ran) where at least one training session each week was a high intensity workout (e.g., sprints, interval, fartlek). Each participant was assigned an alias to use the training mobile application “Strava” (Strava, San Francisco, CA, USA). Participants would upload global positioning system (GPS) tracked run data or manually upload data (e.g., indoor track or treadmill session) to “Strava.” Participants were instructed to upload training data to “Strava” on a daily basis so researchers could ensure compliance and health. The research staff used a shared account to track and log every participant's training sessions. Care was taken by the research staff to contact participants if several days lapsed with no training to check for soreness or injury. Participants had the freedom to dictate how weekly volume was divided for each session. If participants underwent a training session that seemed excessive in volume based on training status then the research team contacted the participant to advise him or her about accumulating fatigue and increasing injury risk potential.

Initially, participants informed the research staff of baseline training volume. Progressive training was induced by increasing weekly volume 5–10% each week whereby participants with lower weekly volume at baseline increased ~10% (e.g., 32 km to 35 km; 20 mi to 22 mi), and participants with higher weekly volume at baseline increased ~5% (e.g., 113 km to 119 km; 70 miles to 74 miles). Mesocyles lasted 4 weeks where training volume increased across weeks 1–3 while week 4 was a recovery week where participants would repeat week 1 volume within that mesocycle. Therefore, volume was increased weeks 1–3, 5–7, and 9, and weeks 4 and 8 were recovery weeks. Week 10 was considered a taper leading into post testing where participants reduced volume to week 1 values (~50% reduction).

### Supplementation and food logs

Supplementation included either placebo pills or whey protein powder. Participants in the PLA were instructed to consume two pills following a training session and ingest two pills before bed on workout days. On non-workout days, participants in PLA were instructed to consume two pills between meals and two pills before bed to emulate PRO. Participants in the whey protein group (PRO) were instructed to consume protein by mixing one scoop of protein powder with 350–500 ml of water. Similarly, on workout days PRO was instructed to consume one scoop immediately following a workout and another scoop before bed. On non-workout days, PRO was instructed to consume one scoop breakfast and lunch or lunch and dinner and one scoop before bed to mitigate catabolic intervals throughout the day based on muscle protein synthesis following protein ingestion (30, 31). Adherence was determined by having participants return empty supplementation canisters every 3 weeks which was met by each participant. The placebo was a sugar pill containing <1.0 g of carbohydrate and no other macronutrients, while one scoop of whey protein powder (Milk Specialties Global; Eden Prairie, MN, USA) provided an additional 120 kcal comprised of the following macronutrients: (a) 1.5 g total fat, (b) 4 g total carbohydrate, (c) 25 g protein, (d) 5.6 g total branched chain amino acids (2.8 g leucine), (e) 11.9 g total essential amino acids, and (f) 13.2 g total non-essential amino acids. Thus, PRO was consuming an additional 240 kcal and 50 g of protein per day through supplementation while PLA did not consume additional nutrients. White plastic canisters were utilized to blind participants to group assignment, and participants were instructed to not converse with peers regarding supplementation due to confounding nature of pills vs. powder. While measures were taken to limit unblinding participants to supplementation, we cannot guarantee blinding was maintained and this is ultimately a limitation to the study. As noted earlier, participants were informed that either supplement may be beneficial based on the sparse chronic protein supplementation literature in endurance athletes.

Food logs were filled out by participants during weeks 1, 5, and 10 to gain perspective on eating habits. Participants were given detailed instructions on how to determine food proportions and thoroughness of meal description needed for accurate assessment. Each participant entered their daily intake via “MyFitnessPal” mobile application (MyFitnessPal, Inc., Baltimore, MD, USA) and submitted spreadsheets constructed by “MyFitnessPal” detailing daily consumption. Four food logs (2 weekdays [Mon-Fri] and 2 weekend days [Sat and Sun]) were submitted by each subject at each time point for a total of 12 food logs. Participants were instructed not to add supplementation to their food logs. Once the study was completed, protein supplementation macronutrients were added manually to the existing totals for PRO (nothing was added to PLA), and then averaged to represent participants' eating habits.

### Statistical analyses

Data are reported throughout as mean ± standard deviation (SD) values. Prior to statistical analyses, all variables were tested for normality using Shapiro-Wilk tests with an alpha level set at *p* < 0.05.

Self-reported macronutrient intake was not normally distributed and certain time points did not pass Levene's test for homogeneity of variances. Forced independent samples *t*-tests for absolute and relative macronutrient intake at each time point were purposefully performed on these measures to fully elucidate any possible differences between groups.

Training data was not normally distributed and certain time points did not pass Levene's test for homogeneity of variances. A (2 × 10) repeated measures ANOVA was utilized where group (PLA, PRO) and time (week 1–week 10) were analyzed. A Huynh-Feldt correction was used if sphericity was violated. Forced independent samples *t*-tests were also purposefully performed on these measures to fully elucidate any possible differences between groups.

Monocyte levels were not normally distributed, and white blood cell levels did not pass Levene's test for homogeneity of variances; however, these variables were analyzed using parametric statistics given the majority of blood data was normally distributed and contained homogenous variances. Body composition, mitochondrial assessment, and 5 km TT were normally distributed and passed the assumption for homogeneity of variances using Levene's tests. A 2 × 2 repeated measures ANOVA was utilized for blood measures, body composition, mitochondrial assessment, and 5 km TT where group (PLA, PRO) and time (Pre, Post) were analyzed. If a significant interaction was identified then pairwise comparisons were utilized for within and between group differences. Pearson correlation coefficients were calculated for mitochondrial capacity and VO_2_peak to determine which variable had a greater relationship with 5 km TT performance. ANCOVAs were also performed on all measures to control for baseline and were found to be inconsequential to the results and therefore are not presented. All of the aforementioned statistical analyses were performed using SPSS version 24.0 (Chicago, IL, USA), and alpha level was set at *p* < 0.05. Effect sizes and 95% confidence intervals were calculated for body composition, mitochondrial assessment, and 5 km TT in Microsoft Excel (Redmond, CA, USA) using the formula for Cohen's d: [(PLA delta mean–PRO delta mean)/pooled SD] where delta refers to post–pre value for each participant. Effect sizes were noted if “large” (d ≥ 0.80).

5 km TT performance was the variable we were most interested in. Thus, power was calculated based on this variable. Hansen et al. (22) reported an effect size of *d* = 0.914 (extrapolated from provided means and standard error) for improvements in 4-km run time following 7 days of whey protein and carbohydrate supplementation. Using this effect size with a power of 80% indicated that 40 total participants, 20 in each group, would be needed for appropriate statistical power. We sought to achieve this number, however had difficulty in recruiting due to rigor of training program, individual training season and plans, and initial running experience.

## Results

### Baseline characteristics

Participant and baseline characteristics can be found in Table 1. There were not significant differences between groups for VO_2_peak (*p* = 0.698) or 5 km TT performance (*p* = 0.166). One female participant in PRO did not complete the post 5 km TT yielding a sample size of eight for this variable. Sex comparisons were considered, but ultimately removed due to small sample size. Of note, independent *t*-tests conducted between sexes on the delta scores for body composition metrics, mitochondrial assessment, or time trial performance did not reveal significant differences between sexes (*p* > 0.05). VO_2_peak showed a significant, strong correlation with Pre 5 km TT (*r* = −0.850; *p* < 0.001) and Post 5 km TT (*r* = −0.881; *p* < 0.001) (*data not shown*).

**Table 1:** Baseline characteristics between treatments.

	**PLA**	**PRO**	***p*-value**
Sample Size	8	9	–
Male: Female	5:3	5:4	–
Age (years)	28 ± 10	33 ± 7	0.231
Height (cm)	173 ± 7	169 ± 8	0.273
Mass (kg)	72.7 ± 8.0	65.9 ± 11.8	0.187
VO2_peak_ (ml/kg/min)	52.6 ± 9.6	54.4 ± 8.7	0.698
5 km Time Trial (min:sec)	25:03 ± 3:08	22:30 ± 3:50	0.166

*Data are presented as mean ± SD. PLA, placebo group; PRO, protein group. 5 km TT n = 8 for both groups. Notably, there are no differences between groups for mass, VO_2_peak, or 5 km TT*.

### Self-reported macronutrient intakes

Self-reported macronutrient data for participants is presented in Table 2. One subject from PLA did not return a food log for week 10. As a result, a sample size of seven was utilized for this time point. As stated previously, forced independent *t*-tests were utilized to detect any possible differences between groups. Relative (g/kg) and absolute (g) carbohydrate (CHO) intake was not different between groups at weeks 1, 5, 10, or overall (average of week 1, 5, and 10). Relative fat intake was significantly different between groups during week 1 and overall (*p* < 0.05).

**Table 2:** Macronutrient consumption throughout training.

		**Week 1**		**Week 5**		**Week 10**		**Overall**	
		**PLA**	**PRO**	**PLA**	**PRO**	**PLA**	**PRO**	**PLA**	**PRO**
CHO	g	230 ± 47	253 ± 93	231 ± 24	245 ± 65	230 ± 42	252 ± 78	228 ± 34	250 ± 77
	g/kg	3.2 ± 0.8	3.9 ± 1.2	3.2 ± 0.5	3.8 ± 1.0	3.3 ± 0.5	4.0 ± 1.2	3.2 ± 0.6	3.9 ± 1.1
Fat	g	66 ± 14	83 ± 22	77 ± 13	83 ± 21	76 ± 15	88 ± 20	73 ± 12	85 ± 19
	g/kg	0.9 ± 0.2	1.3 ± 0.3^#^	1.1 ± 0.2	1.3 ± 0.4	1.1 ± 0.2	1.4 ± 0.4	1.0 ± 0.2	1.3 ± 0.3^#^
Protein	g	79 ± 20	131 ± 22^#^	84 ± 23	134 ± 24^#^	86 ± 13	130 ± 22^#^	84 ± 17	132 ± 21^#^
	g/kg	1.1 ± 0.2	2.0 ± 0.4^#^	1.2 ± 0.3	2.1 ± 0.3^#^	1.2 ± 0.2	2.0 ± 0.3^#^	1.2 ± 0.2	2.1 ± 0.3^#^
Energy	kcal	1828 ± 293	2287 ± 602	1950 ± 216	2264 ± 457	1946 ± 281	2319 ± 487	1904 ± 227	2290 ± 495
	kcal/kg	25.4 ± 4.9	35.2 ± 8.6^#^	27.1 ± 2.7	35.3 ± 7.9^#^	28.1 ± 3.3	36.5 ± 8.0^#^	26.6 ± 3.6	35.7 ± 7.8^#^

*Data are presented as mean ± SD. PLA, Placebo group (n = 8), PRO, Protein group (n = 9), CHO, carbohydrate, kcal, kilocalories. Forced independent samples t-tests were conducted to determine all possible differences. During week 10 PLA n = 7*.

“#” *= Significant difference between groups where p < 0.05. Notably, absolute kcal approached significance weeks 1, 5, 10, and overall (0.05 < p < 0.10)*.

Relative and absolute protein consumption was significantly different during weeks 1, 5, 10, and overall. The PRO group consumed ~50 more grams of protein compared to the PLA group (*p* < 0.05), and nearly double the relative amount of the PLA group (*p* < 0.05).

Relative kilocalorie intake was significantly different between groups at weeks 1, 5, 10, and overall. Absolute kilocalorie intake approached significance weeks 1, 5, 10, and overall (0.05 < *p* < 0.10). The protein group consumed nearly 400 more kilocalories overall compared to the placebo group which came primarily from the increased protein intake.

### Run training

**Figure 1:**
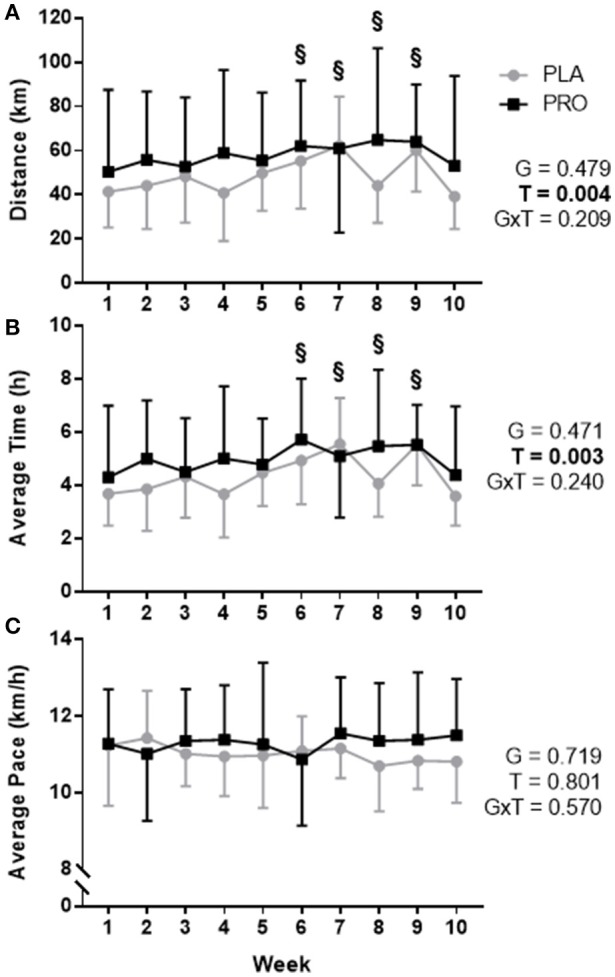
Training data represented as mean ± SD. “G” = group effect *p*-value, “T” = time effect *p*-value, “G×T” = group × time interaction *p*-value. **(A)** Average distance (km) run per week for the placebo group (PLA; gray) and protein group (PRO; black). There was not a significant group by time interaction or group effect (*p* > 0.05), however, there was a time effect (*p* < 0.05) whereby pairwise comparisons determined a significant difference between week 1 and weeks 6, 7, 8, and 9 (“§”, *p* < 0.05). **(B)** Average hours spent running per week for each group. A significant time effect existed (*p* < 0.05) whereby pairwise comparisons determined differences between week 1 and weeks 6, 7, 8, and 9 (“§”, *p* < 0.05). **(C)** Average pace for each group was not significantly different (*p*>0.05). Volume was increased weeks 1–3, 5–7, and 9, and weeks 4 and 8 were recovery weeks. Week 10 was a taper leading into post testing. Forced independent *t*-tests were conducted at each time point to determine any possible group differences and revealed no significant differences (*p* > 0.05).

Training data are presented in Figure 1. An increase in weekly running distance was demonstrated through a repeated measures ANOVA (time *p* = 0.004, group p 0.479, and group × time *p* = 0.209). Pairwise comparisons, independent of group, revealed differences between week 1 (46.2 ± 28.7 km) and week 6 (59.0 ± 25.6 km, *p* = 0.005), week 7 (61.6 ± 30.7 km, *p* = 0.018), week 8 (55.1 ± 33.2 km, *p* = 0.016), and week 9 (62.2 ± 22.3 km, *p* = 0.006) demonstrating an increase in training volume (Figure 1A).

Forced dependent samples *t*-tests revealed PLA significantly increased training volume from week 1 (41.5 ± 16.2 km) to peak training volume–week 7 (62.3 ± 22.2 km; *p* < 0.001). Likewise, PRO significantly increased training volume from week 1 (50.5 ± 37.1 km) to peak training volume–week 8 (64.8 ± 41.6 km; *p* = 0.024). There were no between-group differences based on the ANOVA, and forced independent samples *t*-tests demonstrated no significant differences between groups for distance ran during each week (*p* > 0.05).

Similar differences were seen for average time spent training. An increase in weekly time spent training was observed (time *p* = 0.003, group *p* = 0.471, and group × time *p* = 0.240). Pairwise comparisons, independent of group, demonstrated training time differences between week 1 (4.01 ± 2.08 h) and week 6 (5.35 ± 1.99 h, *p* = 0.002), week 7 (5.31 ± 2.01 h, *p* = 0.020), week 8 (4.81 ± 2.31 h, *p* = 0.009), and week 9 (5.53 ± 1.46 h, *p* = 0.004) demonstrating an increase in time spent training (Figure 1B). There was not a group difference based on the ANOVA, and forced independent samples *t*-tests demonstrated no significant differences between groups for time spent training during each week (*p* > 0.05).

Average pace was not significant based on the ANOVA or significantly different between groups at any week using forced independent samples *t*-tests (Figure 1C). A summary of training data can be found in Supplementary Table S1. Complete training data is organized by each cohort: Fall—Supplementary Table S2, Spring—Supplementary Table S3.

### Blood measurements

No significant interactions or main effects were found between groups for white blood cells, neutrophils, monocytes, red blood cells, or hemoglobin. There was not a significant group × time interaction or time effect; however, there was a significant group effect for lymphocytes (*p* = 0.018) where PLA values were higher than PRO values independent of time. Data are not shown—see Supplementary Table S4 for complete blood measurement data.

### Body composition

**Figure 2:**
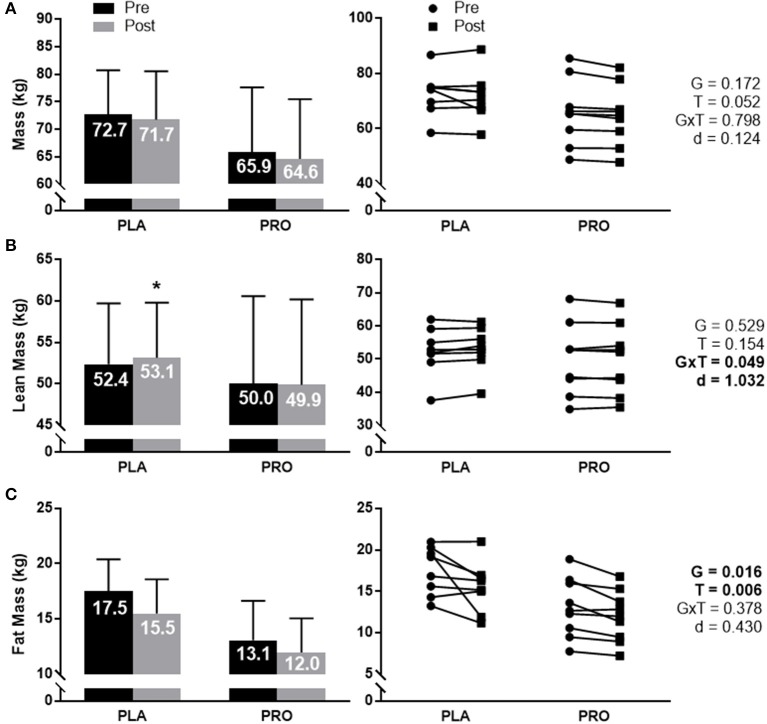
Body composition data represented as mean ± SD. “G” = group effect *p*-value, “T” = time effect *p*-value, “G×T” = group × time interaction *p*-value, “d” = Cohen's d-value. **(A)** The left plot represents average mass (kg) in the placebo (PLA) and protein (PRO) groups at Pre (black bars) and Post (gray bars). The right plot represents individual participant data points for each group from Pre (circle) to Post (square). A time effect approached significance (*p* = 0.052). **(B)** The left plot represents average lean mass (kg) in each group. A significant interaction was found (*p* = 0.049). Pairwise comparisons determined a significant increase from pre to post for PLA (“*” *p* < 0.05). Cohen's d-value also demonstrated a large effect between groups (*d* = 1.032). The right plot represents individual participant data points for each group. **(C)** The left plot represents average fat mass (kg) in each group. A significant group effect was demonstrated (*p* = 0.016) showing greater fat mass in PLA than PRO. A significant time effect was also found (*p* = 0.006) showing a reduction in fat mass from Pre to Post. The right plot represents individual participant data points for each group.

Body mass (Figure 2A) approached significance for a time effect (*p* = 0.052), however there was not a significant group × time interaction, group effect, or meaningful effect size. Average value and standard deviation at pre for PLA (72.7 ± 8.0 kg) and PRO (65.9 ± 11.8 kg), and at post for PLA (71.7 ± 8.8 kg) and PRO (64.6 ± 10.8 kg). Ninety five percent confidence interval for the mean at pre for PLA (66.0–79.4 kg) and PRO (56.8–74.9 kg), and at post for PLA (64.4–79.1 kg) and PRO (56.3–72.9 kg).

Total lean mass (Figure 2B) demonstrated a significant group × time interaction (*p* = 0.049) and large effect size (*d* = 1.032). Pairwise comparisons showed a significant increase from Pre to Post for PLA (*p* = 0.024), but not for PRO. Pairwise comparisons did not demonstrate significant group differences at Pre or Post. Average value and standard deviation at pre for PLA (52.4 ± 7.3 kg) and PRO (50.0 ± 10.6 kg), and at post for PLA (53.1 ± 6.7 kg) and PRO (49.9 ± 10.3 kg). Ninety five percent confidence interval for the mean at pre for PLA (46.3–58.5 kg) and PRO (41.9–58.1 kg), and at post for PLA (47.6–58.7 kg) and PRO (41.9–57.8 kg).

Fat mass (Figure 2C) did not show a significant group × time interaction or meaningful effect size, but did show a significant group effect (*p* = 0.016) where PLA possessed more fat mass than PRO. A time effect was also observed (*p* = 0.006) whereby both groups decreased fat mass from Pre to Post. Average value and standard deviation at pre for PLA (17.5 ± 2.9 kg) and PRO (13.1 ± 3.6 kg), and at post for PLA (15.5 ± 3.1 kg) and PRO (12.0 ± 3.1 kg). Ninety five percent confidence interval for the mean at Pre for PLA (15.1–19.9 kg) and PRO (10.3–15.8 kg), and at Post for PLA (12.9–18.1 kg) and PRO (9.6–14.3 kg).

### Non-invasive mitochondrial capacity assessment using NIRS

**Figure 3:**
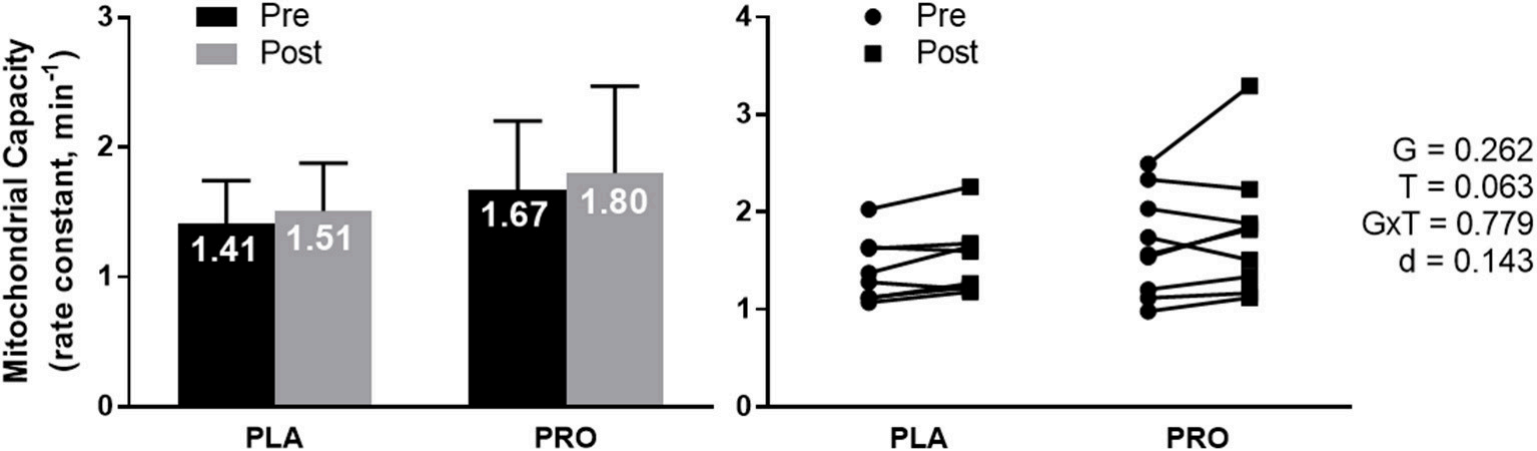
Mitochondrial capacity data represented as mean ± SD. “G” = group effect *p*-value, “T” = time effect *p*-value, “G×T” = group × time interaction *p*-value, “d” = Cohen's d-value. The left plot represents mitochondrial capacity (rate constant, min^−1^) in the placebo (PLA) and protein (PRO) groups at Pre (black bars) and Post (gray bars). A time effect (Post>Pre; *p* = 0.063) approached significance. The right plot represents individual participant data points for each group from Pre (circle) to Post (square).

Mitochondrial capacity assessment using Near-Infrared Spectrometry (NIRS) approached a significant time effect (*p* = 0.063) where both groups improved mitochondrial capacity from Pre to Post. There was not a significant group effect, group × time interaction, or meaningful effect size (Figure 3). Average value and standard deviation at pre for PLA (1.41 ± 0.33 min^−1^) and PRO (1.67 ± 0.53 min^−1^), and at post for PLA (1.51 ± 0.37 min^−1^) and PRO (1.80 ± 0.67 min^−1^). Ninety five percent confidence interval for the mean at Pre for PLA (1.13–1.69 min^−1^) and PRO (1.26–2.08 min^−1^), and at Post for PLA (1.20–1.82 min^−1^) and PRO (1.29–2.32 min^−1^).

Independent of supplementation, there was not a significant correlation between Pre mitochondrial capacity and Pre 5 km TT (*r* = −0.390; *p* = 0.136), Post mitochondrial capacity and Post 5 km TT (*r* = −0.278; *p* = 0.297), Pre mitochondrial capacity and change in 5 km TT (*r* = 0.344; *p* = 0.192), or change in mitochondrial capacity and change in 5 km TT (*r* = −0.177; *p* = 0.511) (*data not shown*).

### 5 km time trial performance

**Figure 4:**
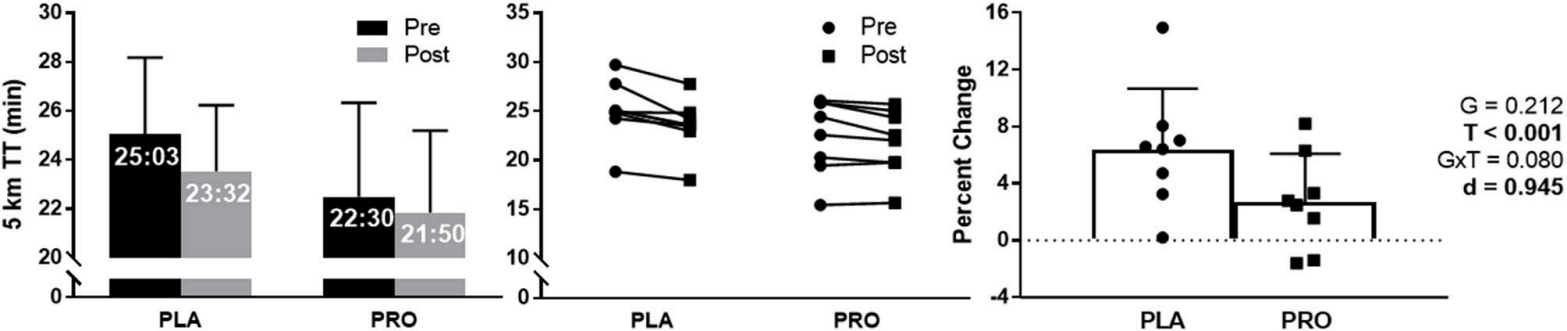
5 km TT data represented as mean ± SD. “G” = group effect *p*-value, “T” = time effect *p*-value, “G×T” = group × time interaction *p*-value, “d” = Cohen's d-value. The left plot represents average time to run 5 km in the placebo (PLA) and protein (PRO) groups at Pre (black bars) and Post (gray bars). The middle plot represents individual participant data points for each group from Pre (circle) to Post (square). The right plot represents average percent change from Pre to Post for PLA (circle) and PRO (square). There was a significant time effect from Pre to Post (*p* < 0.001) demonstrating an improvement in time to completion. The group × time interaction approached significance (*p* = 0.080), and the Cohen's d-value also demonstrated a large effect between groups (*d* = 0.945).

5 km TT performance approached a significant group × time interaction (*p* = 0.080), and demonstrated a large effect size between groups (*d* = 0.945). There was a significant time effect (*p* < 0.001) showing both groups improved 5 km TT performance from Pre to Post; however, there was no significant group effect. PLA improved 5 km TT performance 1min and 31 s, where PRO improved by 40 s (Figure 4). Additionally, PLA improved 5 km TT performance by 6.4% and PRO only improved by 2.7%. Average value and standard deviation at pre for PLA (25:03 ± 3:08 min:sec) and PRO (22:30 ± 3:50 min:sec), and at post for PLA (23:32 ± 2:42 min:sec) and PRO (21:50 ± 3:21 min:sec). Ninety five percent confidence interval for the mean at pre for PLA (22:26–27:40 min:sec) and PRO (19:17–25:42 min:sec), and at post for PLA (21:17–25:47 min:sec) and PRO (19:02–24:39 min:sec).

## Discussion

Our findings refute the hypothesis that protein supplementation would facilitate better improvements in body composition and 5 km TT performance compared to placebo supplementation/run training alone. Surprisingly, PLA outperformed PRO in several metrics. First, PLA demonstrated a large effect size and significant 0.7 kg increase in lean body mass where PRO experienced a miniscule change (−0.1 kg). 5 km TT performance indicated a large effect and approached significance shown through a 6.4% improvement in PLA and only a 2.7% improvement in PRO. These results corroborate and expand on work by Macdermid et al. (32) who determined that a high protein diet for 7 days impaired time trial performance in cyclists. Similarly, Witard et al. (33) reported a “possible” (inferential statistics) attenuation in time trial performance after a block of high-intensity training lasting one week. Given there were no baseline or training differences, and PRO consumed ~1.0 g/kg more protein on average vs. PLA, 10 weeks of whey protein supplementation while run training does not appear to be beneficial for improving endurance performance or body composition.

The training program utilized was designed to maximize outcomes and minimize injury. Positive outcomes have been shown using a linear model of training by strictly increasing volume (34, 35), however to maximize performance outcomes high intensity training should also be performed (36). Furthermore, tapering/recovery has been shown to be effective for performance (37). Herein, we sought to optimize training by instructing participants to increase weekly volume ~5–10%, perform at least one high intensity training session per week, and training weeks were interspersed with recovery weeks at a 3:1 ratio of progressive training to recovery throughout training. We found this training design suitable given that no participants ceased participation due to injury. What is unknown however, is if this method of training is superior to others. Research on periodization for running athletes is sparse, and performance metrics are often not comparable [e.g., 5 km vs. 42.2 km (marathon) TT].

Mitochondrial volume and function is particularly important for endurance athletes given the high metabolic demands associated with endurance exercise. Using NIRS we observed a tendency for mitochondrial capacity to increase with training (time *p* = 0.063). With an adequate sample size we speculate this finding would be significantly different with training based on prior literature where mitochondrial volume and function improve (38). Interestingly, Breen et al. (39) demonstrated that following 90 min of cycling, post-exercise ingestion of CHO (25 g) with whey protein (10 g) does not further augment mitochondrial protein synthesis rates compared to CHO ingestion alone. Our chronic data agree with these acute observations in that meaningful training adaptations in mitochondrial capacity are not enhanced with protein supplementation.

Similar to the present findings, there is literature supporting that running can increase lean body mass. For instance, Dolgener et al. (34) observed increases in fat-free mass following running as well as decreases in body fat percentage. Overend et al. (40) reported that the sum of skinfolds was less following run training signifying a decrease in fat mass and/or increase in lean mass. Other studies have noted after endurance training that body weight remains unaltered (19, 35), although these studies analyzed total weight and did not differentiate between lean and fat mass. Given the large eccentric component of running, it is possible run training increases lean body mass in concert with decreases in fat mass. What is difficult to discern, however, is the increase in lean mass in PLA but not PRO. Furthermore, PLA improved 5 km (3.11 miles) TT performance 1 min 31 s while PRO improved only 40 s. Clearly, run training improves running ability; however, protein supplementation in addition to run training does not seem beneficial and may even prevent run training performance adaptations. It is important to note, while not statistically significant, there was a 2 min 33 s difference between groups in Pre 5 km TT where PLA was slower on average which indicates PLA may have had more room for 5 km TT improvement. Notwithstanding, the relative improvement in 5 km TT performance was nearly 4% higher in PLA, and the two fastest participants in PRO completed the 5 km TT slower following training.

A possible explanation for lower 5 km TT improvement and a decrease in lean body mass in PRO is difficult to determine without mechanistic data from skeletal muscle biopsies. We speculate these findings could be related to aspects of amino acid metabolism. Amino acid transport proteins are elevated in endurance trained individuals (41), and it is well established endurance exercise transiently increases amino acid oxidation (42, 43). Howarth et al. (44) determined the enzyme activity of the rate-limiting reaction in BCAA oxidation is attenuated following endurance training which would combat the increased amino acid oxidation. However, other studies have demonstrated amino acid metabolism is altered under nutrient provision, specifically dietary protein supplementation. For instance, protein supplementation increases BCAA/leucine oxidation at rest, during, and following moderate intensity endurance exercise (8, 45, 46). We speculate the lower improvement in 5 km TT and lack of lean mass change in PRO may be related to an adaptation involving increased, or metabolic fuel preference for, amino acid oxidation at rest and during exercise that would occur under above-average amounts of protein consumption. This phenomenon, in turn, may prevent optimal skeletal muscle responses to training and interfere with performance adaptations.

Certain limitations to the current study should be noted. First, self-reported CHO intake among both groups was low and could have dampened performance improvements in both groups. Importantly, however, CHO intake was not statistically different between groups and would therefore affect both groups similarly. Moreover, the macronutrient intake data were self-reported and could have been underreported; however, careful instruction on logging was given to participants and several days were used to determine macronutrient intake. Notably, both groups consumed adequate amounts of dietary protein potentially masking the ergogenic effect of protein supplementation. This study was designed though to examine the potential benefit of consuming additional dietary protein and not to determine the effect of inadequate amounts of dietary protein. Also, select subjects were elite runners and engaged in very high training volumes which resulted in a high amount of training variability, especially seen in PRO during recovery weeks. This is due in part to the relatively small sample size as well as difficulty in perfect adherence to running prescription. In spite of these limitations, this is the longest study to our knowledge providing protein supplementation during progressive endurance training and examining body composition, performance, and variables related to mitochondrial capacity.

## Conclusions

We demonstrate protein supplementation is not an effective strategy during progressive run training shown through no changes in lean body mass and dampened improvement in TT performance in PRO compared to PLA. These preliminary findings suggest chronic protein supplementation is likely not anabolic or ergogenic in runners. Importantly, protein supplementation did not improve performance compared to run training alone (i.e., consuming placebo). Past literature does suggest, however, acute protein supplementation seems beneficial in altering biomarkers related to improved recovery (13) or an alternative fuel to meet metabolic demands (8), especially when glycogen levels are low (45, 47). Practically, our data in concert with other research findings suggest that protein supplementation seems appropriate during endurance exercise (i.e., marathon or century) or during short periods of recovery (i.e., multi-stage races lasting several days), but not throughout training when an adequate amount of dietary protein is already being consumed (i.e., 1.2–1.5 g/kg).

## Author contributions

PR and MDR conceived the research question. MDR procured materials. All authors recruited and collected data. PR, MDR, and HK analyzed the data. PR and PM conducted statistical analyses. PR wrote the manuscript. MDR provided revisions following initial manuscript draft. All authors revised, edited, and approved the final manuscript.

### Conflict of interest statement

The authors declare that the research was conducted in the absence of any commercial or financial relationships that could be construed as a potential conflict of interest.

## Supplementary material

Supplementary Table S1 Training volume for each participant throughout the study.

Supplementary Table S2 and S3 Daily training volume for each participant throughout the study.

Supplementary Table S4 Blood cell differential data.
